# Retracted: Label-Free Quantitative Mass Spectrometry Reveals a Panel of Differentially Expressed Proteins in Colorectal Cancer

**DOI:** 10.1155/2019/7821495

**Published:** 2019-01-29

**Authors:** BioMed Research International

BioMed Research International has retracted the article titled “Label-Free Quantitative Mass Spectrometry Reveals a Panel of Differentially Expressed Proteins in Colorectal Cancer” [1]. As noted on PubPeer, Figures 5(a) and 5(b) show signs of figure duplication in the Western Blots, where the ERP29 row, lanes 1-6, in Figure 5(a) is the same as the TMP3 row, lanes 2-7 in Figure 5(b), though the former is stretched horizontally.

We were unable to contact the authors. The Editorial Board recommended retracting the article.
